# ESAP plus: a web-based server for EST-SSR marker development

**DOI:** 10.1186/s12864-016-3328-4

**Published:** 2016-12-22

**Authors:** Piyarat Ponyared, Jiradej Ponsawat, Sissades Tongsima, Pusadee Seresangtakul, Chutipong Akkasaeng, Nathpapat Tantisuwichwong

**Affiliations:** 1 Department of Biology, Faculty of Science, Khon Kaen University, Khon Kaen, 40002 Thailand; 2 Department of Computer Engineering, Faculty of Engineering, Khon Kaen University, Khon Kaen, 40002 Thailand; 3 National Center for Genetic Engineering and Biotechnology (BIOTEC), Pathum Thani, 12120 Thailand; 4 Department of Computer Science, Faculty of Science, Khon Kaen University, Khon Kaen, 40002 Thailand; 5 Department of Plant Science and Agricultural Resources, Faculty of Agriculture, Khon Kaen University, Khon Kaen, 40002 Thailand

**Keywords:** Expressed sequence tags (ESTs), Simple sequence repeats (SSRs), EST-SSR development pipeline, Bioinformatics

## Abstract

**Background:**

Simple sequence repeats (SSRs) have become widely used as molecular markers in plant genetic studies due to their abundance, high allelic variation at each locus and simplicity to analyze using conventional PCR amplification. To study plants with unknown genome sequence, SSR markers from Expressed Sequence Tags (ESTs), which can be obtained from the plant mRNA (converted to cDNA), must be utilized. With the advent of high-throughput sequencing technology, huge EST sequence data have been generated and are now accessible from many public databases. However, SSR marker identification from a large in-house or public EST collection requires a computational pipeline that makes use of several standard bioinformatic tools to design high quality EST-SSR primers. Some of these computational tools are not users friendly and must be tightly integrated with reference genomic databases.

**Results:**

A web-based bioinformatic pipeline, called EST Analysis Pipeline Plus (ESAP Plus), was constructed for assisting researchers to develop SSR markers from a large EST collection. ESAP Plus incorporates several bioinformatic scripts and some useful standard software tools necessary for the four main procedures of EST-SSR marker development, namely 1) pre-processing, 2) clustering and assembly, 3) SSR mining and 4) SSR primer design. The proposed pipeline also provides two alternative steps for reducing EST redundancy and identifying SSR loci. Using public sugarcane ESTs, ESAP Plus automatically executed the aforementioned computational pipeline via a simple web user interface, which was implemented using standard PHP, HTML, CSS and Java scripts. With ESAP Plus, users can upload raw EST data and choose various filtering options and parameters to analyze each of the four main procedures through this web interface. All input EST data and their predicted SSR results will be stored in the ESAP Plus MySQL database. Users will be notified via e-mail when the automatic process is completed and they can download all the results through the web interface.

**Conclusions:**

ESAP Plus is a comprehensive and convenient web-based bioinformatic tool for SSR marker development. ESAP Plus offers all necessary EST-SSR development processes with various adjustable options that users can easily use to identify SSR markers from a large EST collection. With familiar web interface, users can upload the raw EST using the data submission page and visualize/download the corresponding EST-SSR information from within ESAP Plus. ESAP Plus can handle considerably large EST datasets. This EST-SSR discovery tool can be accessed directly from: http://gbp.kku.ac.th/esap_plus/.

**Electronic supplementary material:**

The online version of this article (doi:10.1186/s12864-016-3328-4) contains supplementary material, which is available to authorized users.

## Background

Microsatellites or simple sequence repeats (SSRs) are small sequence motifs comprising 1–6 base pairs (bp) in length that are highly conserved in the genome [1]. SSRs are one of the most discriminatively powerful molecular markers because of their co-dominant inheritance, wide genomic distribution, hyper variable, locus specificity, and high reproducibility [2]. Traditional genomic SSR marker identification that directly isolates these markers from a genome is generally time-consuming and labor-intensive. A new method to isolate SSR markers utilizes a motif search over a database of expressed sequence tags (ESTs) is generally called the EST-SSR marker development. This EST-SSR approach is faster, more efficient and more economical than the traditional approach [3].

An EST is generated by a single-pass (from 5′ or 3′ end) sequencing of a clone randomly selected from cDNA libraries [4]. Using ESTs researchers have been able to gather insightful information from their corresponding transcribed genes in various genetic studies, e.g., gene structure identification, alternative splicing detection, and valuable source of EST-SSR markers [1]. Previous studies demonstrated that EST-SSR markers are more useful than genomic markers because ESTs are specifically derived from coding region of genes, which are suitable as functional markers [3, 5, 6]. Developing good EST-SSR markers require high quality ESTs, but often their qualities vary as shown in many public EST databases. The ESTs from such databases may consist of sequences with low quality, unannotated, and redundant [7].

To improve the biological information of ESTs both in the yield and quality, multi-step procedure is required to quality control ESTs (EST cleaning), clustering and assembly, match ESTs to pertinent databases (database matching), and annotate the results (structural and functional annotation) [8]. Presently, several bioinformatic tools for SSR marker development are available. These computational tools must be utilized together in a sequential manner as: pre-processing, EST clustering, SSR mining and primer designing. No complete bioinformatic pipelines that incorporate these steps into one EST-SSR development software suite.

EST sequences represent only a short portion of their corresponding mRNA (approximately 100–800 bp). These derived EST sequences are error sensitive during their sequencing processes. Therefore, the pre-processing step must be carried out to reduce the overall noise in EST data in order to improve the efficacy of the subsequent analyses [8]. First, short EST sequences and unknown nucleotides (N) greater than 5% of the read length need to be removed [9]. A computational tool that can address this problem is, however, not currently available. Second, an EST may be contaminated with a part of its vector sequences, which must be removed. Many software tools were developed to detect and remove such vectors including Cross_match [10], Lucy2 [11], VecScreen [12], Vector cleaning [13], and SeqClean [14]. Most of the tools prefer FASTA format as their inputs. Only few tools accept raw input data in both FASTA and trace file formats. SeqClean is highly recommended as a standard protocol for removing vectors from ESTs as well as other genomic sequences from public database [14]. Finally, any low complexity regions and repetitive elements in the ESTs could cause false sequence clustering and assembly. Thus, these repetitive elements must be masked by a tool such as MaskerAid [15] or RepeatMasker [16].

ESTs are highly redundant and they need to be grouped together to reduce redundancy. TGICL is a tool for clustering EST sequences based on a stringent pairwise comparison between two input EST sequences. The ESTs in these clusters contain significant common regions and they will be assembled to produce consensus sequences [17]. Another popular technique is CD-HIT-EST [18] that first sorts input ESTs by their lengths. These lengths are used to represent different clusters whose members contain EST sequences with high similarity score of sequence alignment. The advantage of CD-HIT-EST is the ultrahigh speed allowing this technique to handle a dataset with millions of sequences. After the above clustering is done, candidate SSR loci can be identified from the clustered EST sequences. Many bioinformatic tools were proposed including Tandem Repeat Finder (TRF) [19], MISA [20], SSRFinder [7], SSRIT [21], TROLL [22], Sputnik and Modified Sputnik I [23], Modified Sputnik II [24], SciRoKo [25] and RepeatMasker [16]. These tools were developed by using different algorithms to look for putative SSR loci.

Designing primers for the candidate SSR loci is the final step of EST-SSR development. Many online and offline primer designing tools are available to predict SSR primers including Primer3 [26], SSR primer [27], BatchPrimer3 [28] and WebSat [29]. WebSat and BatchPrimer3 provide automatic primer designing for SSR amplification and validation on web. However, WebSat restricts the length of input data to 150,000 characters, while BatchPrimer3 offers an offline version for users to download the software to install on a local server with no restriction on the size of the input [28].

The entire workflow for designing EST-SSR primers must utilize various bioinformatic tools. A batch process/workflow for developing large scale EST-SSR primers requires these tools to be in one single pipeline yet the data formats shared among these tools may not be fully compatible.

In this work, we developed an efficient web-based EST analysis pipeline, called ESAP Plus, to facilitate the batch EST-SSR development. ESAP Plus can automatically generate EST-SSR primers directly from raw EST data. ESAP Plus incorporates bioinformatic scripts and software tools necessary for all four important procedures in the development of EST-SSR primers including 1) pre-processing, 2) clustering and assembly, 3) SSR identification and 4) SSR primer design. Since different tools may offer varying performance depending on their inputs, ESAP Plus also offers users with different software choices to include in the pipeline during the clustering/assembly and SSR identification steps. ESAP Plus was implemented as a web-based bioinformatic pipeline to reduce problems with complicated installation procedures and storage requirements. EST-SSR primer results generated by ESAP Plus are shown and can be download from the web interface with e-mail notification when the automatic task is completed. ESAP Plus can be freely used from http://gbp.kku.ac.th/esap_plus/.

## Methods

### Software architecture

ESAP Plus architecture is based on the three-tier architecture model, containing a user interface, processing logic and database tiers. The user interface tier provides graphical interface of ESAP Plus via a familiar web interface, whereas the processing logic tier carries out bioinformatic tasks in the proposed analysis pipeline. We wrote a set of Perl and bash shell scripts to string all standalone bioinformatic routines together in the proposed pipeline. MySQL server (version 5.5) is used to manage raw EST data, intermediate and final output data produced during the automated processing of ESAP Plus. We also keep the reference database required by both RepBase [30] and UniVec [31] in this MySQL server. The ESAP Plus web interface was implemented using HTML, CSS, PHP (version 5) and Java scripts. All three servers, namely web, computation and database servers, run on an Ubuntu Linux server (Ubuntu 12.04.5 LTS) (GNU/Linux 3.2.0-79-generic x86_64) Intel Xeon® CPU X3440 @ 2.53GHz.

### The construction of ESAP Plus automated pipeline

**Fig. 1:**
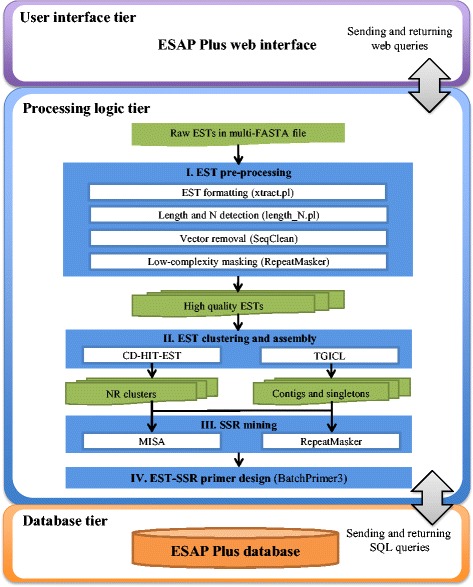
ESAP Plus workflow. The system is based on three-tier architecture including user interface, processing logic, and database tier. Users can interact with the pipeline via the web interface to submit input data and run the pipeline. Processing logic tier consists of multiple tasks which facilitate the analysis of input ESTs. All data and results from the pipeline are stored in the database tier. Users can view and download the stored data though ESAP Plus web interface

ESAP Plus was constructed to identify SSR markers and design the primers from a large EST collection. ESAP Plus includes four main processes to be executed: EST pre-processing, EST clustering and assembly, SSR identification and SSR primer design (Fig. 1). We downloaded and installed standalone version of software tools as well as wrote some shell scripts to handle some tasks as recommended in [9] for these processes. To manage the EST-SSR primer design, we wrote nine in-house shell scripts (Additional file 1). These shell scripts are controlled by the four main core scripts to automate the whole process.

#### EST pre-processing

EST pre-processing is the first process in the proposed pipeline, developed to screen for high-quality ESTs. EST pre-processing has four sub-processes, (1) EST formatting, (2) Length and %N detection, and EST removal, (3) Vector detection and removal and (4) Low-complexity masking. EST formatting module is responsible for converting multiple raw data formats and merge them into a text file with multiple FASTA entries. A Perl script, called xtract.pl, parses raw EST input sequences and converts them into a combined FASTA text file with a “.txt” extension. The second module takes care of screening high quality EST (sequence with ≥100 bp with < 5% of unknown nucleotides) [9]. A Perl script, called length_N.pl, was written to check the length and number of unknown nucleotides in each EST sequence. Low quality sequences will be removed. The third module is called vector detection and removal, which we utilize the SeqClean software [14] and the NCBI UniVec database [31]. SeqClean searches through 3′ or 5′ ends of input EST sequences and removes those regions that are highly similar (>92% identity) to vector, adaptor, primer or linker sequences listed in the UniVec database. The Low-complexity masking module identifies repeat sequences and masks them for removal. To do this, we installed RepeatMasker [16] and included this utility in our pipeline to check the EST sequences from the previous module. RepeatMasker uses RMBLAST (version 2.2.28) to perform the search against the RepBase database [30] for interspersed repeats, repetitive elements and low-complexity DNA sequences. RepeatMasker provides filtering options to identify repetitive elements by users such as DNA source of RepBase, masking and repeat options, or user can use default parameter. The default parameter is set DNA source as human, masking option as repetitive sequence replaced in lowercase, and repeat option as masked interspersed and simple repeats. The EST containing low-complexity region were automatically removed by in-house PHP script of the pipeline.

#### EST clustering and assembly

High quality EST data from the pre-processing stage will be passed to EST clustering and assembly stage. In this part, there are two alternative workflows using two different algorithms, including CD-HIT-EST [18] and TGICL [17]. CD-HIT-EST clusters ESTs and then chooses NR cluster containing non-redundant EST candidates. TGICL produces non-redundant assembled sequences (AS), which are the consensus sequences from both contigs and singletons. The EST clustering cutoff parameters of both CD-HIT-EST and TGICL are adjustable (the default parameter which is set to 95% identity). The resulting non-redundant EST candidates from either CD-HIT-EST or TGICL will be the input of the following SSR mining step.

#### SSR mining

We offer two different algorithms, namely MISA [20] and RepeatMasker [16] for the SSR mining step. MISA can identify both perfect SSRs and compound SSRs (being interrupted by a certain number of bases) [20]. MISA provides users to set parameter to identify SSR or use the default parameter of MISA as follows: a candidate SSR must have at least six di-nucleotide repeats and five tri-, tetra-, penta- and hexa-nucleotide repeats. We also identify candidate SSRs using the RepeatMasker software. The results from both algorithms will be used to design EST-SSR primer pairs.

#### EST-SSR primer design

EST-SSR sequences obtained from SSR mining of both MISA and RepeatMasker will be sent to BatchPrimer3 [28] that utilizes Primer3 core [26] to design primers. To reduce resulting false positive primers, BatchPrimer3 incorporates SSR filtering that uses SSRIT algorithm [21] to select high quality template for primer design. BatchPrimer3 provides users to set parameter for SSR screening and primer design or use default parameter. The default cutoff parameter of SSR screening is set to have at least six di-nucleotide repeats and five tri-, tetra-, penta- and hexa-nucleotide repeats. The default parameters of BatchPrimer3 to primer design are set as follows: 150–300 bp product size, with 18–27 bp of primer size, primer temperature minimum at 57 °C and maximum at 63 °C, primer GC% minimum at 50 °C and maximum at 80 °C. The primer design results along with other intermediate results produced by the proposed pipeline will be stored in the ESAP Plus MySQL database.

### Evaluation data sources

A total of 232,352 ESTs derived from sugarcane (*Saccharum* spp.) from 26 cDNA libraries of SUCEST project [32] were downloaded from GenBank dbEST (retrieved on 19 August 2014). This EST collection was used as raw EST input data to evaluate the performance of ESAP Plus. This experiment was conducted using the default parameters of ESAP Plus to design EST-SSR primer pairs for sugarcane excepted low-complexity masking step that DNA source of RepBase as *Saccharum* spp. was used.

### ESAP Plus database overview

ESAP Plus allows users to submit raw EST data from multiple popular file formats, including “.seq”, “.fasta” and “.txt”, through ESAP Plus web interface. The input file will be submitted to the pipeline, whose name will be automatically converted to a timestamp value. At each computational stage, we append the stage number to the name of each EST sequence name. For instance, if the accession number of an EST input data as specified after the “>” symbol in the header is ABXXXX, the extension “.1” will be appended as “>ABXXXX.1” and “>ABXXXX.2”, if this file is processed in the second stage. Modified EST sequences at each stage are stored in the MySQL database.

The ESAP Plus MySQL database contains the following four interlinked tables: est_user (personal information), est_contact (user contact information), work_queuing (jobs submitted to ESAP Plus) and work_done (completed job and output reports). To save working space and time to download results, we compress all data (zip format) and offer links to download which are valid for one week.

## Results

### ESAP Plus web interface

**Fig. 2:**
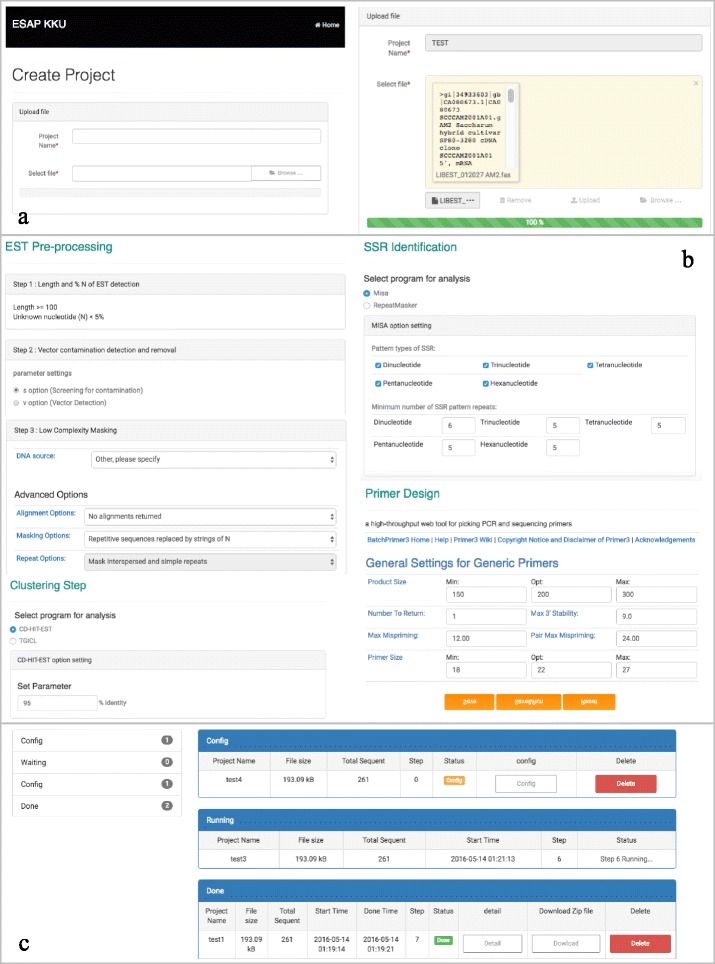
A screenshot of ESAP Plus interface. **a** Upload section: users can upload input data into the pipeline. **b** Software parameters can be configured at all stages of the proposed pipeline. The clustering step provides users to choose between two optional software tools for clustering or/and assembly ESTs, namely CD-HIT-EST and TGICL. It also provides two alternatives for mining SSR by using MISA or RepeatMasker. **c** User page shows the status of all users’ projects such as configuration jobs, running jobs, and completed jobs

The web interface consists of three main sections, home, upload and project. Users must first register through ESAP Plus web interface in the home section. This is required so that a secure space will be allocated for the registered user. Once logged in, users can upload raw EST data using the form in the upload page. When the data is being uploaded, a green bar will indicate the uploading progress (Fig. 2a). Upon completion of data uploading, users can click next button and proceed to the configuration page (Fig. 2b). The ESAP Plus provides various parameters and software options for users for every step in the pipeline. In particular, we offer different software choices in clustering/assembly (CD-HIT-EST vs. TGICL) and SSR mining (MISA vs. RepeatMasker) steps. When the software configuration is done, users can start the automatic process by clicking the run button while the save button is also provided to save the configuration settings. After the completion of the EST-SSR primer design, users will be notified by e-mail. Figure 2c shows a dashboard view showing all statistics, configuration jobs, running jobs and completed jobs.

**Fig. 3:**
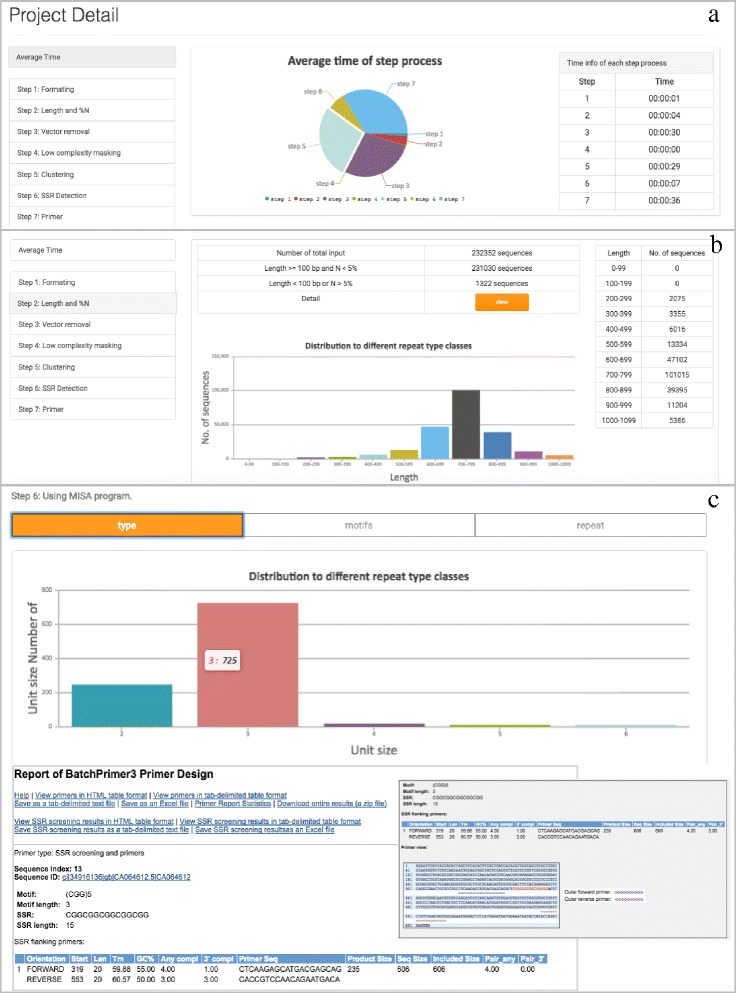
Overview of ESAP Plus result page. ESAP plus offers graphical web interface for users to visualize statistical information during the processing of input ESTs. **a** An overview of a project is shown in pie chart. **b** Users can view detailed information such as length and N detection. **c** Types of SSR, SSR motif as well as repeats are reported with detailed information of each SSR primer, e.g., forward and reverse primers, position, expected product size

The summary view of ESAP Plus allows users to view results in the user page. All intermediate results produced during the computation are collected in the database and can be downloaded through the web interface (Fig. 3) with the pie chart summary report (Fig. 3a). For EST pre-processing, lengths of EST sequences along with unknown nucleotides are shown in the table and bar chart (Fig. 3b). For SSR detection, SSR types, motifs, and repeat numbers are reported. For SSR primer design, the web interface shows the details of SSR motif and SSR length. Forward and reverse primers with starting position, primer lengths and sequences are also reported in this section (Fig. 3c).

### Validation of ESAP Plus workflow

A total of 232,352 sugarcane ESTs from 26 cDNA libraries [32] downloaded from dbEST were analyzed by ESAP Plus using the default parameters. These input EST sequences are larger than or equal to 100 bp. Approximately 1,322 ESTs contain more than 5% of unknown nucleotides, which were removed by our workflow (Table 1). Consequently, 231,030 ESTs were passed to vector detection and removal stage. At this stage, we found 42,594 ESTs contains vectors at the 5′ or 3′ end, and these vectors were removed from the sequences. Only 62 ESTs were vector-contaminated in multiple regions of these EST sequences, which were automatically deleted (Table 1). In addition, 5,194 low-complexity regions in 4,890 EST sequences were masked as indicated by lowercase letters (Table 1). The EST containing low-complexity region were automatically removed.

**Table 1:** Number of intermediate EST results at each ESAP Plus major stage

Number of raw ESTs			232352	
Step 1. Pre-processing				
N > 5%			1322	
Vector trimming			42594	
Vector-contaminated deletion			62	
Low-complexity masking			5194	
Number of high-quality ESTs			226078	
Step 2. Clustering and assembly				
Software options	CD-HIT-EST		TGICL	
Number of NR clusters/ASs	142788		65713	
Total length (bp)	95445342		51256243	
Step 3. SSR identification				
Software options	MISA	RepeatMasker	MISA	RepeatMasker
Number of SSR containing in NR clusters/ASs	9327	25708	5412	14490
Total number of identified SSRs	10240	32021	5951	18526
Number of SSR types				
di-NTRs	2446	1784	1203	884
tri-NTRs	7311	15410	4446	9291
tetra-NTRs	247	2419	159	1341
penta-NTRs	128	2524	77	1355
hexa-NTRs	108	9884	66	5655
An average of 1 SSR/Kbp	9.32	2.98	8.61	2.77
Step 4. Primer design				
Number of successfully designed SSR primer pairs	7850	6272	4613	3783

From the EST pre-processing step, 226,078 EST sequences were identified as high-quality. Then, these high-quality ESTs were clustered or/and assembled by TGICL or CD-HIT-EST. With CD-HIT-EST, 226,078 high-quality ESTs were clustered into 142,788 non-redundant clusters. While TGICL clustered and assemble high-quality ESTs into 65,713 assembled sequences (ASs) (26,340 contigs and 39,373 singletons).

During the SSR mining step, in 142,788 non-redundant clusters by CD-HIT-EST, a total number of 9,327 and 25,708 NR clusters were detected as containing SSR loci by MISA and RepeatMasker, respectively. In 65,713 ASs by TGICL, a total number of 5,412 and 14,490 ASs were found to contain SSR loci by MISA and RepeatMasker, respectively. Despite this, in 9,327 and 25,708 NR clusters, there were 10,240 and 32,021 SSR loci while 5,412 and 14,490 ASs contain 5,951 and 18,526 SSR loci, respectively (Table 1).

Although there are some variations in the distribution of nucleotide repeat types among software sets, trinucleotide repeats were the most abundance of SSR type (Table 1). From MISA detected SSR loci (10,240 and 5,951), ESAP Plus successfully designed 7,850 and 4,613 SSR primer pairs, respectively. From RepeatMasker detected SSR loci (32,021 and 18,526), ESAP Plus successfully designed 6,272 and 3,783 SSR primer pairs, respectively (Table 1).

### The use of SSR markers developed by ESAP Plus

Randomly selected fifteen SSR primer pairs (Additional file 2: Table S1), generated by ESAP Plus, were subjected to SSR amplification reactions by using sugarcane genomic DNA as template. All primer pairs successfully amplified SSRs and detected length polymorphism within 15 sugarcane cultivars (Additional file 3: Figure S1). The polymorphic information content (PIC) values ranged from 0.00-0.93 (Additional file 2: Table S1). An example of SSR primer pair, namely SU018, produced SSR amplicons which their sizes varies between 138-157 bp. The PIC of this primer pair was 0.84 (Additional file 2: Table S1). This result indicated that most of SSR primers produced from ESAP Plus could be used as polymorphic DNA markers suitable for sugarcane genetics and breeding studies.

## Discussion

### Performance and utility of ESAP Plus

We assessed ESAP Plus using 232,352 ESTs. ESAP Plus was able to handle relatively large EST data sets. In terms of software features, we compared ESAP Plus with existing three publicly available EST analysis workflows [33–35] as shown in Table 2. ESAP Plus is the only workflow that provides all four important steps, namely the pre-processing, clustering, SSR identification and primer design stages. Moreover, ESAP Plus allows users to choose different software during the clustering (CD-HIT-EST vs. TGICL) and SSR mining (MISA vs. RepeatMasker) stages. ESAP Plus was designed to automatically process raw EST sequences and produce the designed primers via web interface with no limitation on input sizes. We tuned the default parameters throughout the computational pipeline based on the experiment on a large sugarcane EST dataset. Users can easily adjust most software parameters via the configuration page (Fig. 2b). Users will be notified via e-mail when the analysis is done and the results can be conveniently downloaded from the download page.

**Table 2:** Feature comparisons of publicly available EST analysis pipelines

	Pipeline options					
	Pre-processing	Clustering and assembly	SSR mining	Primer design	Website	Output
PESTAS	Phred	TGICL	-	-	JSP	View on website
	Cross_match					
	SeqClean					
	RepeatMasker					
ESTpass	Cross_match	CAP3	-	-	HTML	View on website
	RepeatMasker	d2_cluster			Java	Download file
ESMP	Cross_match	CAP3	MISA	-	HTML	View on website
	Trimest				CSS	Download file (.rar)
					Java script	
					PHP	
ESAP Plus	Length_N.pl	CD-HIT-EST	MISA	BatchPrimer3	HTML	View on website
	SeqClean	TGICL	RepeatMasker		CSS	Download file (.zip)
	RepeatMasker				Java script	
					PHP	

### Data evaluation and SSR distribution in sugarcane ESTs

ESAP Plus identified that the sugarcane EST sequence data are 97.30% high quality. We noticed that a choice of software tools during EST redundancy removal could be a critical stage. In addition, there are much more differences in the numbers of detected SSR loci between MISA’s and RepeatMasker’s. A number of SSR loci detected by RepeatMasker is three times more than that of MISA’s. Such differences may greatly be influenced by MISA’s detection technique that uses a keyword-tree, while RepeatMasker attempts to align possible SSR patterns from RepBase with the input EST data using Smith-Waterman algorithm [36]. Smith-Waterman offers more relax condition for RepeatMasker to detect more SSR loci than MISA’s. During the primer designing stage, we observed that BatchPimer3 successfully generated more output primer pairs from MISA’s detected SSR loci than that of RepeatMasker. Since BatchPrimer3 performs, by default, SSR filtering prior to designing primers, many SSR loci produced by RepeatMasker could be filtered out by BatchPrimer3’s SSR filtering step.

The trinucleotide repeats were abundant SSR type identified by ESAP Plus. This result is also consistent with the previous sugarcane studies [37] and close evolutionary species such as in maize (*Zea mays* L.), rice (*Oryza sativa* L.), sorghum (*Sorghum bicolor* L.), wheat (*Triticum aestivum* L.) [6] and barley (*Hordeum vulgare*) [20]. The most abundant SSR motif was CCG/CGG that is identical with some cereal species such as barley, wheat, maize, oats, rye, rice, and sorghum [6, 38].

## Conclusions

ESAP Plus is an automated pipeline for developing EST-SSR primers. ESAP Plus is the most comprehensive and flexible software suite for EST-SSR primer development that incorporates all four critical steps, namely, 1) EST pre-processing, 2) clustering and assembly, 3) identifying SSR locus and 4) design SSR primers. The proposed pipeline offers users to choose different tools that might be better fit with their input ESTs for reducing EST redundancy and identifying SSR loci. ESAP Plus is capable of handling large scale design of EST-SSR primers that is needed in most plant genetics studies and breeding programs.

## Availability and requirements

Project name: ESAP Plus.

Project home page: http://gbp.kku.ac.th/esap_plus/.

Operating systems: the software is fully tested on Ubuntu Linux operating system.

Programming languages: Perl, Shell script, PHP, HTML, Java script

Other requirements: Apache HTTP server, Perl interpreter

License: GNU GPL

Any restrictions to use by non-academics: None

The dataset of ESTs supporting the result of this article was published by SUCEST projects [32] and it is accessible from the dbEST repository, [http://www.ncbi.nlm.nih.gov/dbEST/].

## Additional files

Additional file 1: Supplementary materials. Lists of in-house Shell script in ESAP Plus. Nine in-house Shell scripts including: (1) est1_formatting.sh, (2) est2_length_N.sh, (3) est3_vector.sh, (4) est4_lowcom.sh, (5) est5_est_clustering_cdhitest.sh, (6) est5_est_clustering_tgicl.sh, (7) est6_ssr_misa.sh, (8) est6_ssr_repeatmasker.sh, and (9) est7_primer.sh. Format: The above shell scripts were compressed in one zip file. (ZIP 10 kb)

Additional file 2: Table S1. A list of 15 SSR primers used to amplify DNA fragments by PCR reaction with genomic DNAs extracted from 15 sugarcane cultivars. (DOCX 15 kb)

Additional file 3: Figure S1. PCR amplification results of primer SU018 for 15 commercial cultivars of sugarcane (*Saccharum* spp.). (PPTX 894 kb)
