# Supplementary material for: ESAP plus: a web-based server for EST-SSR marker development
Source: BMC Genomics. 2016 Dec 22;17(Suppl 13):1035. doi: 10.1186/s12864-016-3328-4 (PMC5260030; doi:10.1186/s12864-016-3328-4)
Supplement: Supplementary file 2 — A list of 15 SSR primers used to amplify DNA fragments by PCR reaction with genomic DNAs extracted from 15 sugarcane cultivars. (DOCX 15 kb) [file 12864_2016_3328_MOESM2_ESM.docx]

### **Additional file 2**

### Table S1: A list of 15 SSR primers used to amplify DNA fragments by PCR reaction with genomic DNAs extracted from 15 sugarcane cultivars.

| **Primer name** | **Accession number of EST** | **Expected size of SSR (bp)** | **Allele sizes in sugarcane genomic DNA** | **PIC** |
| --- | --- | --- | --- | --- |
| SU001 | AA961302 | 208 | 172-227 | 0.93 |
| SU002 | BQ478955 | 236 | 225-253 | 0.85 |
| SU003 | BQ478920 | 201 | 183-211 | 0.93 |
| SU004 | BQ478953 | 100 | 99-105 | 0.77 |
| SU006 | pss_cl5_contig1 | 278 | 320-323 | 0.23 |
| SU007 | pss_cl163_contig1 | 101 | 94-108 | 0.82 |
| SU008 | BQ536073 | 154 | 130-157 | 0.84 |
| SU009 | BQ536809 | 188 | 145-191 | 0.75 |
| SU014 | CA180670 | 111 | 226-228 | 0.00 |
| SU015 | CA192339 | 167 | 141-172 | 0.65 |
| SU016 | CA186106 | 111 | 209-216 | 0.71 |
| SU017 | CA182156 | 124 | 118-126 | 0.82 |
| SU018 | CA182224 | 149 | 138-157 | 0.84 |
| SU019 | CA182277 | 167 | 152-168 | 0.87 |
| SU021 | CA184826 | 149 | 133-156 | 0.92 |

### Figure S1: PCR amplification results of primer SU018 for 15 commercial cultivars of sugarcane (*Saccharum* spp.)
