# Supplementary material for: ESAP plus: a web-based server for EST-SSR marker development
Source: BMC Genomics. 2016 Dec 22;17(Suppl 13):1035. doi: 10.1186/s12864-016-3328-4 (PMC5260030; doi:10.1186/s12864-016-3328-4)
Supplement: Supplementary file 3 — PCR amplification results of primer SU018 for 15 commercial cultivars of sugarcane (Saccharum spp.). (PPTX 894 kb) [file 12864_2016_3328_MOESM3_ESM.docx]

### **Additional file 3 – Supplementary Figure**


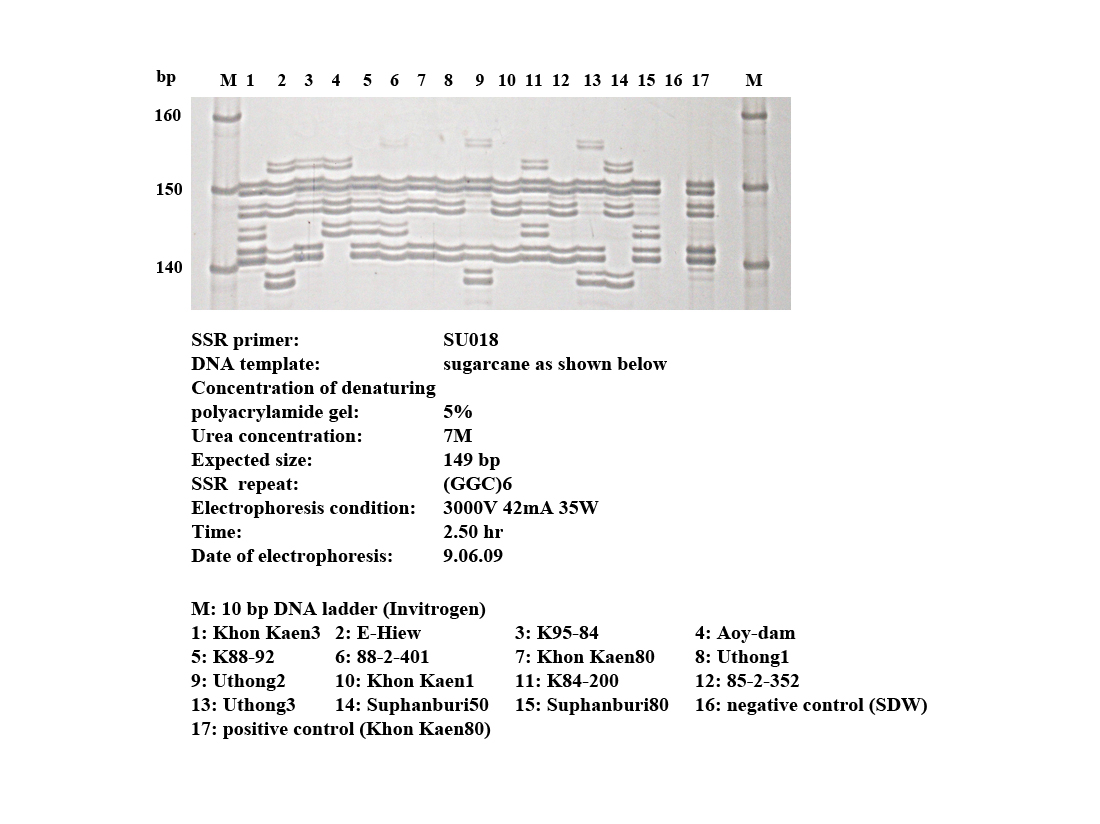


145 bp

142 bp

139 bp

157 bp

154 bp

151 bp

150 bp

147 bp

144 bp

141 bp

138 bp

### Figure S1: PCR amplification results of primer SU018 for 15 commercial cultivars of sugarcane (*Saccharum* spp.)
